# Plasma urotensin-2 level and Thr21Met but not Ser89Asn polymorphisms of the urotensin-2 gene are associated with migraines

**DOI:** 10.1186/s10194-016-0623-z

**Published:** 2016-04-18

**Authors:** Sırma Geyik, Sercan Ergun, Samiye Kuzudişli, Figen Şensoy, Ebru Temiz, Erman Altunışık, Murat Korkmaz, Hasan Dağlı, Seval Kul, Aylin Akçalı, Ayşe Münife Neyal

**Affiliations:** Department of Neurology, Faculty of Medicine, University of Gaziantep, Gaziantep, Turkey; Ulubey Vocational Higher School, Ordu University, Ordu, Turkey; Department of Neurology, Emine-Bahaeddin Nakiboglu Medical Faculty, Zirve University, Gaziantep, Turkey; Neurology Clinics, Medical Park Hospital, Gaziantep, Turkey; Department of Medical Biology, Faculty of Medicine, University of Gaziantep, Gaziantep, Turkey; Division Of Neurology, Turkish Ministry Of Health Siirt State Hospital, Siirt, Turkey; Department of Biostatistics, Faculty of Medicine, University of Gaziantep, Gaziantep, Turkey

**Keywords:** Migraine without aura, Urotensin-2, ELISA, UTS2 gene polymorphisms, Thr21Met, Ser89Asn

## Abstract

**Background:**

Urotensin-II (U-II) is a peptide recognized by its potent vasoconstrictor activity in many vascular events, however the role of urotensin-II in migraine has not been considered yet. The molecular mechanisms and genetics of migraine have not been fully clarified yet, but it is well-known that vascular changes considerably contribute in pathophysiology of migraine and also its complications. The aim of this study was to analyze the plasma U-II levels along with genotype distributions and allele frequencies for UTS2 Thr21Met and Ser89Asn polymorphisms among the patients with migraine without aura (MWoA).

**Methods:**

One hundred eighty-six patients with MWoA and 171 healthy individuals were included in this study. Plasma U-II levels were measured in attack free period. The genotype and allele frequencies for the Thr21Met (T21M) and Ser89Asn (S89N) polymorphisms in the UTS2 gene were analyzed.

**Results:**

Plasma U-II levels were significantly higher in MWoA patients (*p* = 0.002). We detected a significant association between the T21M polymorphism in the UTS2 gene and migraine (53.8 % in patients, 40.4 % in controls, *p* = 0.035), but not with S89N polymorphism (*p* = 0.620). A significant relationship was found between U-II levels and MIDAS score (β = 0.508, *p* = 0.001).

**Conclusion:**

Our study suggests that U-II may play a role in migraine pathogenesis; also Thr21Met polymorphism was associated with the risk of migraine disease. Further studies are needed for considering the role of U-II in migraine pathophysiology and for deciding if UTS2 gene may be a novel candidate gene in migraine cases.

## Background

Migraine is a common neurological disorder that affects approximately 12 % of the population [1]. However, in recent years, it has been suggested that migraines are formed as a result of neuronal vascular event chains triggered by endogenous and/or exogenous factors in people with a genetic predisposition [2, 3]. Many first-degree relatives of migraine patients have a history of migraines, and twin studies conducted show that migraines have a strong genetic component [4–6].

Urotensin-2 (U-II) is a cyclic peptide composed of 11 amino acids that was first isolated from the goby neurosecretory system in 1969 [7]. The human receptor for U-II (hUT2R) is a G-protein coupled receptor (GPR14) [8]. U-II and its receptor (UTR) are found in different tissues such as the central nervous system, peripheral vascular tissues, the heart, and the kidneys [8, 9]. Clark et al. suggested that UII receptor mRNA and choline acetyltransferase exist together in the mesopontine tegmental area [10]. U-II is a vasoactive substance that has a similar peptide structure to somatostatin and is a more powerful vasoconstrictor than endothelin-1 (ET-1) [11]. The vasoconstrictor effect of U-II is 50 times higher on arteries and 10 times higher on veins than ET-1. U-II is thought to have endothelium-dependent vasodilator and endothelium-independent vasoconstrictor effects, and its net effect may depend on the balance between these two individual effects [12]. However, studies have shown that it plays other physiological roles beyond the regulation of vascular tone and cholinergic activity. The urotensinergic system has been shown to be associated with heart failure, hypertension, diabetes, preeclampsia, renal and liver diseases, neurological and psychiatric disorders [13, 14]. U-II is known to be expressed in the brain and spinal cord. It is recognized as a neuro-mediator in the central nervous system [15, 16].

U-II may also play a role in migraine pathogenesis, especially considering its known effects on the central and peripheral nervous system. The U-II gene (UTS2) is located at the 1p36 locus. According to data from the US National Center for Biotechnology Information, more than 60 single nucleotide polymorphisms (SNPs) have been recorded in the human UTS2 gene. Thr21Met (T21M, rs228648) and Ser89Asn (S89N, rs2890565) polymorphisms have been found at high allelic frequencies in Japanese populations [17]; these are the same polymorphisms that were selected for investigation in our study. Although many studies have shown the roles of various genetic factors and polymorphisms in migraine disease, no studies have focused on theT21M and S89N polymorphisms in the UTS2 gene in migraine patients until now.

Therefore, the purpose of this study is to examine the possible relationships between the T21M and S89N polymorphisms in the UTS2 gene and MWoA and to detect the possible role of U-II in the pathogenesis of MWoA by measuring serum U-II levels.

## Methods

### Study population

Study approval was obtained from the Ethics Committee of the Gaziantep University Faculty of Medicine. Informed consent was obtained from all subjects prior to the study. This study examined 186 consecutive patients aged 18–45 years, who were diagnosed as having migraines, had not been on prophylactic treatment for at least 3 months and had least 72 h migraine attack drug-free period before obtaining blood samples in the interictal phase. Since it is thought to have endothelium-dependent vasodilator and endothelium-independent vasoconstrictor effects, and its net effect may depend on the balance between these two individual effects and we don’t know if it has an effect in migraine attacks we decided to obtain the blood samples in the interictal phase from all of the patients.

Migraines were diagnosed according to the ICHD-2 criteria [18] by experienced neurologists in our clinic and then enrolled into the study. For homogenizing the group, we selected only MWoA patients. The control group (*n* = 171) was composed of age and gender matched healthy cases that consented to join to the control group.

Cases with history of diabetes mellitus (fasting blood glucose ≥ 120 mg/dl); hypertension (Blood pressure (BP) ≥ 140/90 mmHg); chronic renal failure; liver cirrhosis; any type of cancer; thyroid diseases; alcohol and substance abuse; chronic neurologic illnesses, including epilepsy, Parkinson’s disease, Huntington’s disease, Alzheimer’s disease, Wilson’s disease, and previous cerebrovascular and cardiovascular diseases, morbid obesity; and any existing infection were excluded from both groups. The medical histories, physical and neurologic examination findings, and body mass indices (BMI) of all cases were recorded. The migraine patients were questioned regarding the disease duration, the type of migraine, the frequency of migraine attacks (for the last 3 months), drugs used, and smoking. The Migraine Disability Assessment Scale (MIDAS) was applied to measure the extent to which migraine headaches decreased the patients’ standard of living. Routine laboratory examinations, including total blood count, serum electrolytes, serum creatinine, blood urea nitrogen (BUN), fasting blood glucose levels, and liver function tests, were performed in all cases. The glomerular filtration rate (GFR) was calculated according to the Modification of Diet in Renal Disease (MDRD) guidelines [19].

### Power analysis

Sample size was estimated using a power calculation based on 0.2 ± 0.6 changes in urotensin between groups. It was estimated that at least 142 participants in each group would be required to detect a significant difference between control and migraine groups at 80 % power level and an alpha error of % 5.

### Blood samples and DNA isolation

Venous blood samples were drawn from the antecubital vein in the morning hours after 12 h of fasting and for at least 72 h without symptomatic migraine medication. The plasma was separated from the blood samples by adding EDTA and centrifuging the samples at 1000 g for 15 min. The plasma samples were then stored at -80 °C until U-II levels were measured. Plasma U-II concentrations were measured using a quantitative sandwich-type enzyme immunoassay UT2 kit (Elx 800 ELISA; Cusabio Biotech, Winooski, VT, USA). Genomic DNA extraction was performed from the plasma-free blood pellets using a standard proteinase K and salt precipitation method. The extracted DNA was stored at -20 °C.

### SNP genotyping

Samples were genotyped for the UTS2 SNPs using a validated TaqMan SNP Genotyping Assay (Applied Biosystems Inc. (ABI), Foster City, CA, USA) that employed predesigned primers and probes for the UTS2 gene SNPs (T21M, rs228648; S89N, rs2890565) (ABI). One allelic TaqMan probe was labeled with a fluorescent FAM dye, and the other was labeled with a VIC dye. For each polymerase chain reaction (PCR), 5 μL of genomic DNA solution (5 ng/μL) was added to an aliquot of 2× TaqMan universal PCR Master Mix, resulting in primer and probe final concentrations of 180 and 40 nM, respectively. The amplification protocol consisted of the following steps: (1) an initial denaturation at 95 °C for 10 min and (2) 40 cycles of denaturation at 95 °C for 15 s and annealing and extension at 60 °C for 1 min, with amplification and fluorescence detection performed using a Qiagen Rotor-Gene Q Real-time PCR system. At least 10 % of the blood samples were run twice in separate assays with a concordance of genotype designation of 100 %.

### Statistical analysis

The results are expressed as either means ± the standard deviations (SDs) or the percentage. Statistical analysis was performed using the Statistical Package for Social Sciences (SPSS) software version 20.0 (Inc. Chicago, IL). The chi-squared test was used to calculate significant differences in the genotype and allele frequencies. The unpaired Student’s t test was used to compare the differences between the mean values of the 2 groups. The effects of the genetic polymorphisms on the risk of SSc were estimated with an odds ratio (OR) and 95 % confidence interval (CI). The haplotype analysis was performed using SHEsis software (http://analysis.bio-x.cn/myAnalysis.php). All of the statistical tests and p values were two-sided, and *p* < 0.05 was considered statistically significant.

## Results

One hundred eighty-six patients diagnosed with MWoA and 171 healthy control subjects were enrolled in this study. No significant differences existed between the two groups in terms of gender distribution, age, BMI, or smoking status. A total of 101 (54.3 %) migraine patients were using migraine attack drugs (triptans, nonsteroidal anti-inflammatory drugs (NSAIDs), paracetamol and combination analgesic). The demographic and laboratory characteristics of the study group are shown in Table 1.

**Table 1:** Demographic, clinical and laboratory characteristics of the patient and control groups

Parameters	Patients (*n* = 186)	Control (*n* = 171)	*p*
Mean age (years)	29.30 ± 5.45	28.77 ± 5.44	0.360
Gender (n, %)			
Female	133 (71.5)	124 (72.5)	0.832
Male	53 (28.5)	47 (27.5)	
BMI (kg/m^2^)	25.94 ± 2.84	26.02 ± 2.86	0.795
Hgb (g/dL)	12.68 ± 0.6	12.66 ± 0.6	0.538
Wbc (×10^3^/mL)	5.9 ± 1.17	4.46 ± 1.17	0.190
Urea (mg/dL)	18.55 ± 4.91	18.60 ± 4.95	0.927
GFR (ml/min)	106 ± 7	101 ± 9	0.206
ESR (mm/h)	4.96 ± 1.44	4.97 ± 1.45	0.929
CRP (mg/L)	0.71 ± 0.3	0.7 ± 0.3	0.725
Smoking (n, %)	41 (22)	38 (22.2)	0.967
Disease duration (years)	6.98 ± 3.87		
Attack frequency (n, %)			
1–5/month	114 (61.3)		
6–10/month	56 (30.1)		
> 10/month	16 (8.6)		
MIDAS score	2.86 ± 1.12		
Vomiting	99 (52.3)		
FF	143 (76.8)		
Mood changes	132 (70.9)		

*BMI* body mass index, *ESR* erythrocyte sedimentation rate, *CRP* C-reactive protein, *GFR* glomerular, *Hgb* hemoglobin, *WBC* white blood cell

*MIDAS* Migraine Disability Assessment Scale, *FF* photophobia and phonophobia

### Plasma urotensin-2 levels by ELISA assay

**Fig. 1:**
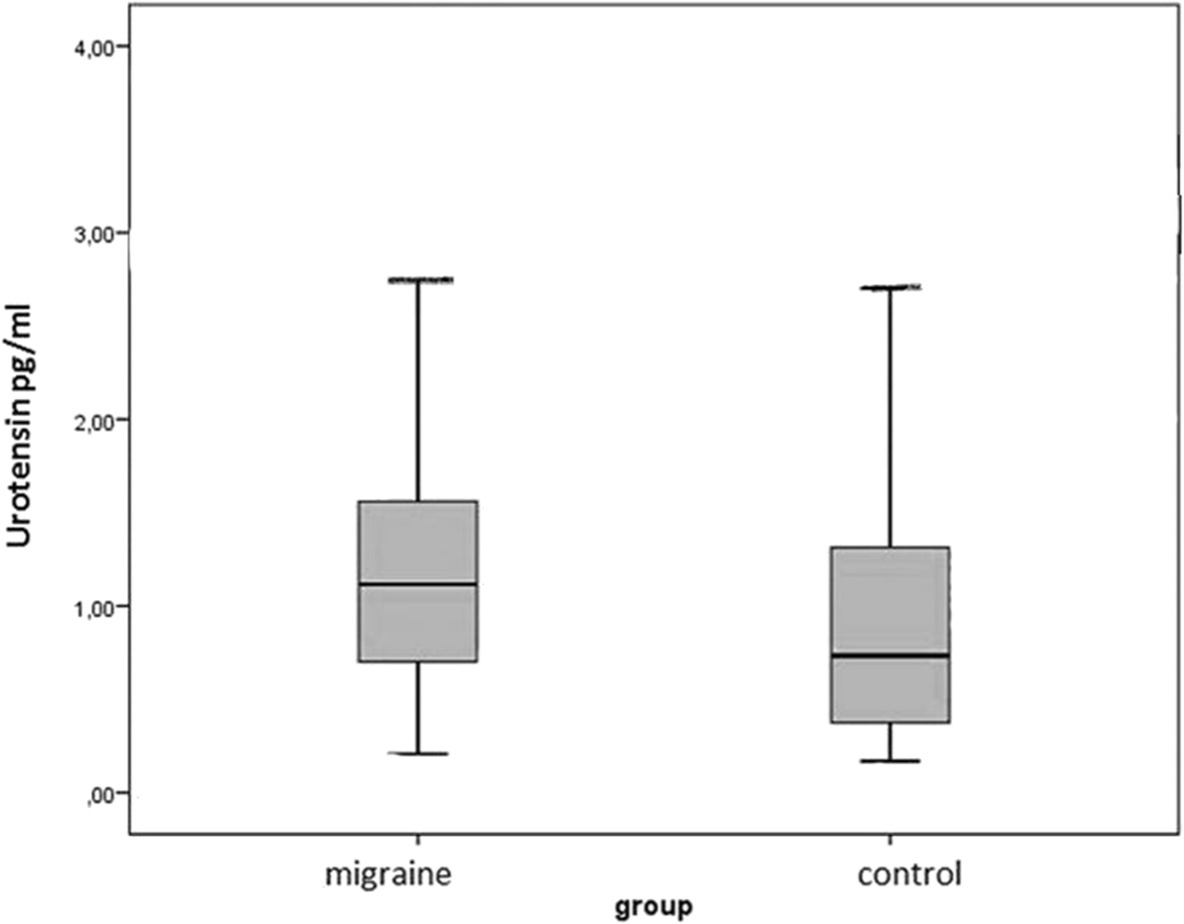
Urotensin-2 levels (pg/mL) in the migraine and control groups

**Fig. 2:**
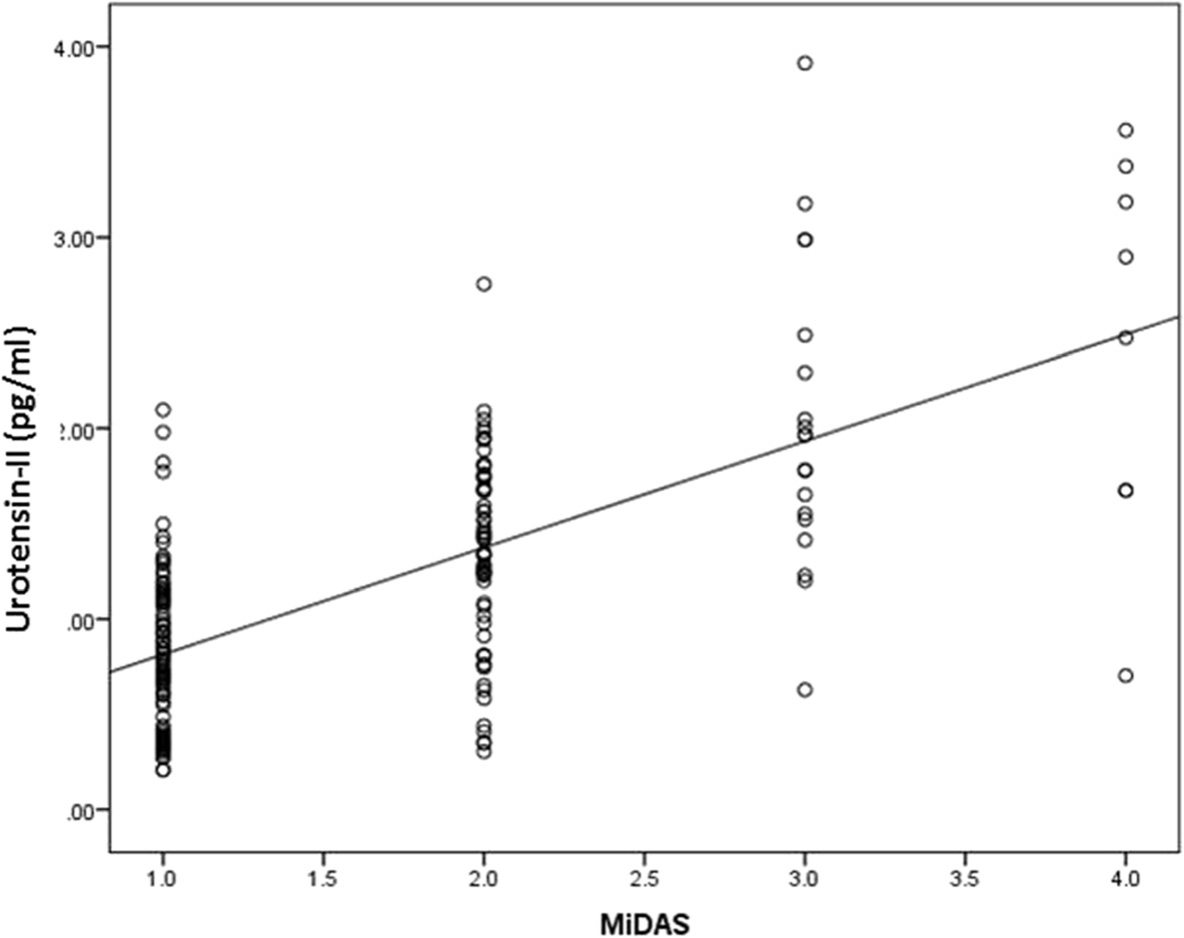
The correlation between U-II levels and MIDAS scores

The mean U-II levels were 1.19 ± 0.69 pg/ml in the migraine group and 0.97 ± 0.7 pg/ml in the control group. U-II plasma levels were significantly higher in the migraine group (*p* = 0.002) (Fig. 1). No differences in U-II levels were found in terms of gender, smoking status, symptomatic medication history in either group (Table 2). Disease duration and attack frequency were not significantly correlated with U-II levels (β:0.48/p:0.657, β: 0.195/p:0.175; respectively). A significant relationship was found between U-II levels and MIDAS score. U-II levels were significantly higher in patients with higher MIDAS scores. A 1 unit increase in the MIDAS score resulted in a 0.508 unit increase in U-II levels (β = 0.508, *p* = 0.001) (Fig. 2).

**Table 2:** Comparison of plasma Urotensin-II levels according to demographic and clinical variables

Demographic and clinical variables	Urotensin (pg/ml)		
	Migraine	Control	*p* value
Smoking	1.15 ± 0.51	0.99 ± 0.52	0.003*
Non-smoking	1.104 ± 0.6	0.82 ± 0.61	0.001*
*p* value	0.965	0.854	
Female	1.19 ± 0.48	0.96 ± 0.62	0.001*
Male	0.98 ± 0.45	0.84 ± 0.48	0.003*
*p* value	0.185	0.832	
Drugs (+)	1,13 ± 0.4	-	
Drugs (–)	1,11 ± 0.51	-	
*p* value	0,981		
Vomiting (+)	1.21 ± 0.6		
Vomiting (–)	1.04 ± 0.51		
*p* value	0.004*		
FF (+)	1.19 ± 0.5		
FF (-)	1.17 ± 0.6		
*p* value	0.967		
Mood (+)	1.28 ± 0.7		
Mood (-)	0.99 ± 0.6		
*p* value	0.001*		

Valuables are expressed as the mean ± SD

Drug (+): Using symtomatic migraine drugs

Drug (-): Not using symptomatic migraine drugs

*FF* photophobia and phonophobia

*p < 0.05

### Genotyping

Genotype and allele frequencies for T21M (rs228648) and S89N (rs2890565) polymorphisms in the UTS2 gene in the patient and control groups are shown in Table 3. We detected a significant association between the T21M polymorphism in the UTS2 gene and migraine but no significant relationship between the S89N polymorphism and migraine in our study (*p* = 0.620). T21T genotype frequency was more prevalent in the control group (34.4 % in the patients compared with 42.1 % in the controls). T21M genotype frequency was more prevalent in the migraine group (53.8 % in the patients compared with 40.4 % in the controls, *p* = 0.035) and the According to these results, patients with theT21M genotype are 1.63 times more likely to become migraine patients than patients with the T21T genotype (OR = 1.63, *p* = 0.035). No significant differences were found in the 21 M polymorphism allele frequencies between the migraine and control groups (*p* = 0.786). Moreover, no significant relationships were found between the MIDAS scores and the T21M (*p* = 0.502) or S89N (*p* = 0.300) polymorphisms in the UTS2 gene in the migraine groups. Finally, no significant relationships were found between the smoking status and the T21M (*p* = 0.885) or S89N (*p* = 0791) polymorphisms in the UTS2 gene in the migraine group. There were insignificant increases in MN, MS and TS haplotype frequencies in migraine patients (Table 4).

**Table 3:** Distributions of the T21M and S89N polymorphisms among the groups

Genotype/Allele	Control (*n* = 171) n (%)	Migraine (*n* = 186) n (%)	p	OR [95 % CI]
T21M				
TT	72 (42.1)	64 (34.4)	Reference	
TM	69 (40.4)	100 (53.8)	0.035	1.63 [1.034–2.571]
MM	30 (17.5)	22 (11.8)	0.559	0.825 [0.433–1.572]
T	213 (62.3)	228 (61.3)	Reference	
M	129 (37.7)	144 (38.7)	0.786	1.043 [0.771–1.411]
HWE	*p* = 0.065	*p* = 0.069		
S89N				
SS	151 (88.3)	161 (86.6)	Reference	
SN	20 (11.7)	25 (13.4)	0.620	1.172 [0.625–2.198]
NN	0 (0.0)	0 (0.0)	-	
S	322 (94.2)	347 (93.3)	0.632	0.862 [0.470–1.582]
N	20 (5.8)	25 (6.7)	Reference	
HWE	*p* =0.417	*p* =0.325		

*OR* odds ratio, *CI* confidence interval, and *HWE* Hardy Weinberg equilibrium

*p < 0.05

**Table 4:** Haplotype distributions of UTS2 gene polymorphisms in migraine patients and controls

Site 1	Site 2	Case (freq)	Control (freq)	p	Odds Ratio [95 %CI]
M	N	24.99 (0.067)	18.45 (0.054)	0.468	1.257 [0.676–2.338]
M	S	119.01 (0.320)	110.55 (0.323)	0.891	0.978 [0.714–1.340]
T	N	0.01 (0.000)^a^	1.55 (0.005)^a^	-	-
T	S	227.99 (0.613)	211.45 (0.618)	0.822	0.966 [0.714–1.307]

^a^Frequencies < 0.03 were ignored in the analysis

### Relationship between genotypes and expression

**Fig. 3:**
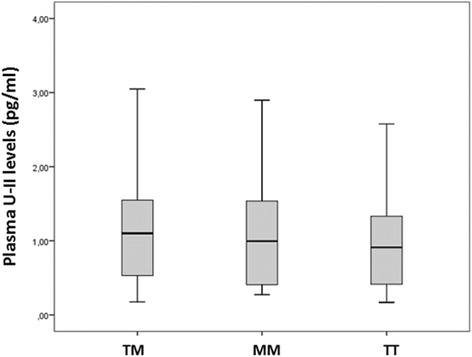
The relationship between plasma U-II protein level and Thr21Met polymorphism in patients

The plasma levels of U-II were tended to be higher without statistical significance in TM and MM genotype (*p* = 0.545). The relationship between plasma U-II protein level and Thr21Met polymorphism in patients was shown in Fig. 3.

## Discussion

Our results revealed a significant elevation of serum U-II levels in migraine patients. Additionally, a significant association of migraine with T21M polymorphism but not with S89N polymorphism of urotensin gene was observed in the present study.

Several peptides have been reported in relation with migraine pathophysiology. The role of calcitonin gene-related peptide (CGRP) in migraine pathogenesis is still under investigation in current researches [20, 21]. Pituitary adenylate cyclase-activating polypeptide and substance P (SP) had craniocervical vasodilatation, plasma protein extravasation, peripheral and central sensitization effects in migraine pathogenesis [22]. Serum levels of vasoactive intestinal peptide (VIP); a marker of parasympathetic nervous system, was found to be increased in chronic and episodic migraineurs in attack free period [23]. Diverse results for neuropeptide Y (NPY); a marker of sympathetic nervous system with long-lasting vasoconstrictor effects in cerebral circulation, was reported in migraineurs [22].

U-II and its receptor have many functions, including the regulation of behavior and neuroendocrine activities, cardiovascular tone control, motor activity, the sleep-wake cycle, and hypothalamic-pituitary-adrenal axis control [14, 24–26]. U-II plasma concentrations are low in healthy individuals [13]. Previously some studies have measured higher UT2 peptide levels in various metabolic and cardiovascular diseases such as; chronic heart failure, acute myocardial infarction, hypertension and diabetes mellitus [27–29].

Considerably increased risk of ischemic events, in various organs including brain has already been documented in migraine [30]. Currently the exact mechanisms of that relation between migraine and stroke are not understood entirely. Distinct mechanisms were proposed for migraine-related ischemia that occurs either during the attack or attack-free periods. Additionally, it is not clear yet if stroke is a consequence of migraine or both of the conditions occur due to another shared pathological course. In this case, a genetic link may be responsible for influencing the course and variability of the consequences of migraine [31]. Nevertheless, endothelial dysfunction was proposed as one of the main paths in this association [32, 33].

U-II is a vasoactive peptide with ubiquitous effects in various human body tissues. It has been suggested to have endothelium-dependent vasodilator and endothelium-independent vasoconstrictor effects, with its net effect depending on the balance between these two individual effects [12]. Yet in general, U-II is known to have a powerful vasoconstrictor effect.

As mentioned above, since U-II has dual effect on endothelium through distinct pathways and we don’t have data if it has an effect in migraine attacks or not, the blood samples were obtained in the interictal phase in all of the cases. Therefore, the significantly higher levels of plasma U-II in the present study present only the U-II status in the attack-free period that may be a limitation of the present study. Understanding the effects of U-II on the attacks is yet to be waiting for further investigation. However, we believe that the stated concerns about the dual effect of U-II should be taken under consideration also in further research that aim to investigate the plasma U-II effects in migraine attacks.

We found a significant positive relation between the MIDAS scores and plasma U-II levels in the present study. This finding may indicate that the severity of migraine may be influenced by plasma U-II and in turn it may have a bad effect in quality of life of the cases.

Many first-degree relatives of migraine patients have a history of migraine, and twin studies showed that migraine have a strong genetic component [3–5]. Genetic factors may cause a tendency (predispose) to have migraine attacks. Despite increasing number of studies suggesting a role for genetic factors in the pathogenesis of migraine, the responsible genes have not yet been determined. Despite, neuronal vascular event chains triggered by endogenous and/or exogenous factors in people with a genetic predisposition, it is now believed that neuronal dysfunction is the possible primary reason in the pathophysiology of the disease and vasodilation and vasoconstriction phases are probably epiphenomena [34, 35].

Non-familial migraines can be considered as polygenic when the diversity of both the number and severity of attacks and the duration of the attacks are considered. Various genes were supposed to be involved in migraine pathophysiology. Our results indicate that a significant association exists between the T21M polymorphism and migraine. The T21M genotype patients were 1.63 times more likely to become migraine than patients with the T21T genotype in the present study. Amino acid change upon Thr21Met polymorphism may affect protein folding efficiency or structure. By this way, differently folded protein may be exposed to protein degradation processes more or less than natively folded protein. Also, some amino acid sequences form signal for degradation. With this amino acid change, degradation signal may be created and this causes U-II level to decrease. Moreover, newly formed Met codon on mRNA may provide potential translation starting point for ribosome, although it is a low probability. So, this causes a truncated U-II protein product. All these possibilities may explain U-II level changes upon Thr21Met variant presence. Also, we revealed that the plasma U-II levels tended to be higher in cases with Thr21Met polymorphism as shown in Fig. 3. However the difference couldn’t reach a statistically significant level.

Several publications have indicated a possible relationship between UTS2 gene polymorphisms and hypertension, diabetes mellitus, Behçet’s disease, and systemic sclerosis [17, 36–38]. Recently, genome-wide studies have provided new insights for genes associated with migraine disease (the ion channel gene, TRPM8, FHL5, ASTN2, and LRP1 [39] but UTS2 gene was not one of them. Since, we believe that it is worthy to evaluate this vasoactive peptide in migraine pathophysiology since vascular changes predominantly take place in migraine cases in both attack and attack-free periods.

## Conclusion

Our study suggests that U-II may play a role in migraine pathogenesis, also Thr21Met polymorphism was associated with the risk of migraine disease. Further studies are needed for considering the role of U-II in migraine pathophysiology and for deciding if UTS2 gene may be a novel candidate gene in migraine cases.

## Abbreviations

BMI: body mass index
CGRP: calcitonin gene-related peptide
CI: confidence interval
ET-1: endothelin-1
FHM: familial hemiplegic migraine
GPR14: G-protein coupled receptor
hUT2R: human receptor for U-II
ICHD-3: International Classification of Headache Disorders
MDRD: Modification of Diet in Renal Disease
MIDAS: Migraine Disability Assessment Scale
MWoA: migraine without aura
NPY: neuropeptide Y
NSAID: nonsteroidal anti-inflammatory drug
OR: odds ratio
S89N: Ser89Asn
SD: standard deviation
SP: substance P
T21M: Thr21Met
U-II: urotensin-2
UTR: U-II receptor
VIP: vasoactive intestinal peptide
